# Arrhythmic events pertinent with antidepressants: a Bayesian disproportional analysis mining the FDA Adverse Event Reporting System database

**DOI:** 10.3389/fpsyt.2025.1637471

**Published:** 2025-09-29

**Authors:** Shan Cao, Zhuoshan Huang, Wenjie Yang, Saijia Yang, Xi Chen, Xujing Xie

**Affiliations:** ^1^ Department of Cardiology, The Third Affiliated Hospital of Sun Yat-sen University, Guangzhou, Guangdong, China; ^2^ Clinical Research Center, The Third Xiangya Hospital, Central South University, Changsha, Hunan, China; ^3^ Department of Epidemiology and Statistics, School of Public Health, Medical College, Zhejiang University, Hangzhou, Zhejiang, China; ^4^ School of Health, Brooks College, Sunnyvale, CA, United States

**Keywords:** arrhythmic events, antidepressants, Bayesian disproportional analysis, FAERS database, pharmacovigilance

## Abstract

**Objective:**

The aim of this study was to investigate pharmacovigilance (PV) and make pairwise comparisons on arrhythmic events among antidepressants from the US Food and Drug Administration Adverse Event Reporting System (FAERS).

**Methods:**

Records regarding antidepressants treating depression and major depression from the first quarter of 2015 to the third quarter of 2023 documented in the FAERS database were harvested. The primary endpoint of this study was PV for arrhythmic events, including QT prolongation/Torsades de Pointes (TdP), atrial fibrillation (AF), heart block, and ventricular arrhythmia. The secondary endpoints comprised the pairwise comparisons on constituent ratio and severity of outcomes of drugs of interest on the above four diseases.

**Result:**

Ultimately, 746,507 records were eligible for analysis. PV for QT interval prolongation/TdP was identified for citalopram [proportional reporting ratio (PRR) = 2.13, 95% confidence interval (CI): 1.89 to 2.40, reporting odds ratio (ROR) = 2.14, 95% CI: 1.90 to 2.40, IC = 0.98, 95% CI: 0.80 to 1.12], escitalopram (ROR = 1.72, 95% CI: 1.52 to 1.96, IC = 0.71, 95% CI: 0.51 to 0.86), fluoxetine (ROR = 1.39, 95% CI: 1.21 to 1.60, IC = 0.43, 95% CI: 0.21 to 0.60), and quetiapine (ROR = 1.58, 95% CI: 1.30 to 1.91, IC = 0.63, 95% CI: 0.31 to 0.85). In terms of AF, PV was detected in citalopram (ROR = 1.82, 95% CI: 1.44 to 2.30, IC = 0.78, 95% CI: 0.41 to 1.05), escitalopram (ROR = 1.34, 95% CI: 1.03 to 1.74), sertraline (ROR = 1.32, 95% CI: 1.07 to 1.64, IC = 0.35, 95% CI: 0.01 to 0.59), and fluoxetine (ROR = 1.68, 95% CI: 1.32 to 2.13, IC = 0.68, 95% CI: 0.29 to 0.95). With regard to heart block, PV was detected in citalopram (ROR = 1.37, 95% CI: 1.05 to 1.80) and mirtazapine (ROR = 1.40, 95% CI: 1.03 to 1.90). Regarding ventricular arrhythmia, PV was detected in citalopram (ROR = 1.55, 95% CI: 1.19 to 2.02, IC = 0.58, 95% CI: 0.15 to 0.88), escitalopram (ROR = 1.51,95% CI: 1.16 to 1.97, IC = 0.54, 95% CI: 0.12 to 0.85), and quetiapine (PRR = 2.39, 95% CI: 1.75 to 3.25, ROR = 2.39, 95% CI: 1.75 to 3.26, IC = 1.17, 95% CI: 0.67 to 1.54).

**Conclusion:**

Citalopram and escitalopram [classified as selective serotonin reuptake inhibitors (SSRIs)] exhibited the strongest correlations with arrhythmic occurrences. Quetiapine [classified as a second-generation antipsychotic (SGA)] demonstrated higher risk and worse prognosis on QT prolongation/TdP and ventricular arrhythmic events. Venlafaxine and duloxetine [classified as serotonin-norepinephrine reuptake inhibitors (SNRIs)] did not show any PV of any arrhythmia and had lower risks and a lower degree of adverse events compared with the rest. Certainly, more head-to-head related studies are merited.

## Introduction

World Health Organization (WHO) surveillance data indicated that depression affects an estimated 280 million people globally, corresponding to 3.8% of the world’s population, with an 18% increase in case burden observed from 2005 to 2015 (1). Elevated adolescent depression prevalence and associated suicide rates intensify aging-driven demographic pressures, potentially impeding national socioeconomic progress.

Initial depression management relied on tricyclic antidepressants (TCAs). The 1986 advent of fluoxetine—a potent selective serotonin reuptake inhibitor (SSRI)—catalyzed targeted monoaminergic therapies, later expanding to serotonin-norepinephrine reuptake inhibitors (SNRIs) with enhanced safety. The US Food and Drug Administration (FDA) subsequently approved second-generation antipsychotics (SGAs) in 2008 as adjunctive treatment for major depressive disorder (MDD) following the demonstrated clinical efficacy.

Emerging evidence indicates antidepressant-related pro-arrhythmic risks, particularly SSRI-associated QT prolongation and potential Torsades de Pointes (TdP) in a dose-dependent manner (2, 3). However, a recent retrospective cohort analysis of treatment-naive patients showed no increased arrhythmia incidence with SSRIs (4). This evidence discordance complicates risk–benefit assessments in depression management.

Given that the latent manifestation of arrhythmia pathogenesis necessitates longitudinal surveillance beyond conventional trial frameworks, we leveraged real-world evidence from the FDA Adverse Event Reporting System (FAERS) database. Our Bayesian disproportionality analysis of antidepressant-related arrhythmic adverse events (AEs) delivers pharmacovigilance (PV) insights essential for optimizing depression therapeutics.

## Methods

### Data sources

Data from the first quarter of 2015 to the third quarter 2023 were downloaded from https://open.fda.gov/data/downloads/. Subsequently, the data were converted into a comma-separated value (csv) format using R studio 4.3.2 and Python Spyder 5.0 since the primary files were encoded with java script.

### Definition of adverse event of interest

In the FAERS, AEs are described using System Organ Class (SOCs) and preferred terms (PTs) from the Medical Dictionary for Regulatory Activities (MedDRA version 27.0). In order to examine the association between antidepressants and cardiac AEs, the Standardized MedDRA Queries (SMQs) were screened using a “narrow” version. The AEs of interest in this study were cardiac arrhythmias, which were categorized as follows: QT prolongation/TdP, atrial fibrillation (AF), heart block, and ventricular arrhythmia. The grouping of PTs according to the characteristics of AEs is presented in **Supplementary Tables 1**-**4**.

### Definition of antidepressants of interest

Using dispensing frequency data for England’s top 10 antidepressants (data from Statista - The Statistics Portal for Market Data, Market Research and Market Studies), we categorized medications by prescription prevalence. Concomitantly, we queried the FAERS database to evaluate the utilization of antidepressants exclusively in patients with depression/MDD, stratifying agents identically. Per Canadian Network for Mood and Anxiety Treatments (CANMAT) guidelines (5), TCAs were recommended as second-line recommendations. Hence, TCAs were not enrolled in the subsequent analysis. Despite its well-documented proarrhythmic potential, quetiapine was included in this study for systematic comparative analysis owing to its established efficacy in MDD and bipolar depression, for which it is strongly recommended by clinical guidelines (6). Thus, eight antidepressants met inclusion criteria.

### Statistical analysis

In this study, the proportional reporting ratio (PRR), the reporting odds ratio (ROR), and the Bayesian Confidence Propagation Neural Network (BCPNN) were employed as statistical indicators.

The ROR stands out for its ability to synthesize results from multiple studies on adverse drug reactions while accounting for heterogeneity among studies, such as design differences, sample characteristics, and adverse reaction definitions, offering crucial references for drug development and clinical practice (7). The PRR stands out for its greater specificity compared to ROR. BCPNN performs well in integrating multi-source data and cross-validation (8). The triangulation of these methodologies enhances the robustness of our conclusions by providing mutual validation.

These metrics quantified the relationship between a drug and an adverse reaction and showed a positive correlation with it, thus indicating the presence of a PV signal when the statistical threshold corresponding to the metric is exceeded. The calculation equation and criteria are listed in **Supplementary Table 5**.

All data analyses were performed using the STATA 18.0 MP software.

## Results

According to the frequency of records, eight antidepressants, namely, citalopram, escitalopram, fluoxetine, sertraline, venlafaxine, duloxetine, mirtazapine, and quetiapine, were designated as the main focus of the study. A total of 746,507 eligible AE reports were subjected to analysis.

### Descriptive analysis

There are a total of 2,671 records of QT prolongation/TdP, a total of 689 records of AF, a total of 655 records of heart block, and a total of 616 records of ventricular arrhythmia. Clinical characteristics are presented in **Table 1**. Patients with arrhythmic events among antidepressants were older, with a higher proportion of female patients, seriousness, and hospitalization (**Table 1**).

**Table 1:** Baseline characteristics of arrhythmic event related to drug treatments in patients with depression in FAERS database.

Characteristic	QT prolonged/TdP	Atrial fibrillation (AF)	Heart block	Ventricular arrhythmia
Total (n)	2671	689	655	616
Age (years)^∥^	53 (37, 69)	66 (58, 75)	64.5 (53, 72)	52.5 (38, 66)
Weight (kg)^∥^	68 (57,78.5)	87.1 (70, 100.4)	77 (59.02, 91.0)	71.20 (57, 86.0)
Sex (%)				
male	656 (24.6)	322 (46.7)	158 (24.1)	146 (23.7)
female	1718 (64.3)	324 (47)	430 (65.6)	407 (66)
unknown	297 (11.1)	43 (6.2)	67 (10.2)	63 (10.2)
Region of reporting (%)				
United States	496 (18.6)	330 (47.8)	166 (25.3)	159 (25.8)
Other countries	1957 (81.0)	293 (42.7)	482 (73.7)	449 (73)
Unknown	11 (0.4)	66 (9.5)	7 (1)	8 (1.2)
Seriousness (%)II				
Serious	2660 (99.6)	676 (98.1)	652 (99.5)	604 (98.1)
No	11 (0.4)	13 (1.9)	3 (0.5)	12 (1.9)
Hospitalization (%)				
Yes	1552 (58.2)	502 (72.9)	499 (76.2)	406 (65.9)
No	207 (7.7)	39 (5.7)	21 (3.2)	33 (5.4)
Unknown	912 (34.1)	148 (21.4)	135 (20.6)	177 (28.7)
Death (%)				
Yes	220 (8.2)	62 (9.0)	31 (4.7)	116 (18.8)
No	474 (17.8)	103 (14.9)	97 (14.8)	86 (14.0)
Unknown	1977 (74.0)	524 (76.1)	527 (80.5)	414 (67.2)
Disabling (%)				
Yes	67 (2.5)	27 (3.9)	39 (6.0)	22 (3.6)
No	507 (19.0)	105 (15.2)	83 (12.6)	108 (17.5)
Unknown	2097 (78.5)	557 (80.9)	533 (81.4)	486 (78.9)
Life-threatening (%)				
Yes	854 (32.0)	102 (14.8)	109 (16.6)	262 (42.5)
No	255 (9.5)	78 (11.3)	92 (14.1)	59 (9.6)
Unknown	1562 (58.5)	50 (73.9)	454 (69.3)	295 (47.9)

^ǁ^ shown as median (IQR); II includes: death, life-threatening, requires inpatient hospitalization or prolongation of existing hospitalization, results in persistent or significant disability/incapacity, congenital anomaly/birth defect, and other serious important medical event.

### Disproportionality analysis

**Figure 1:**
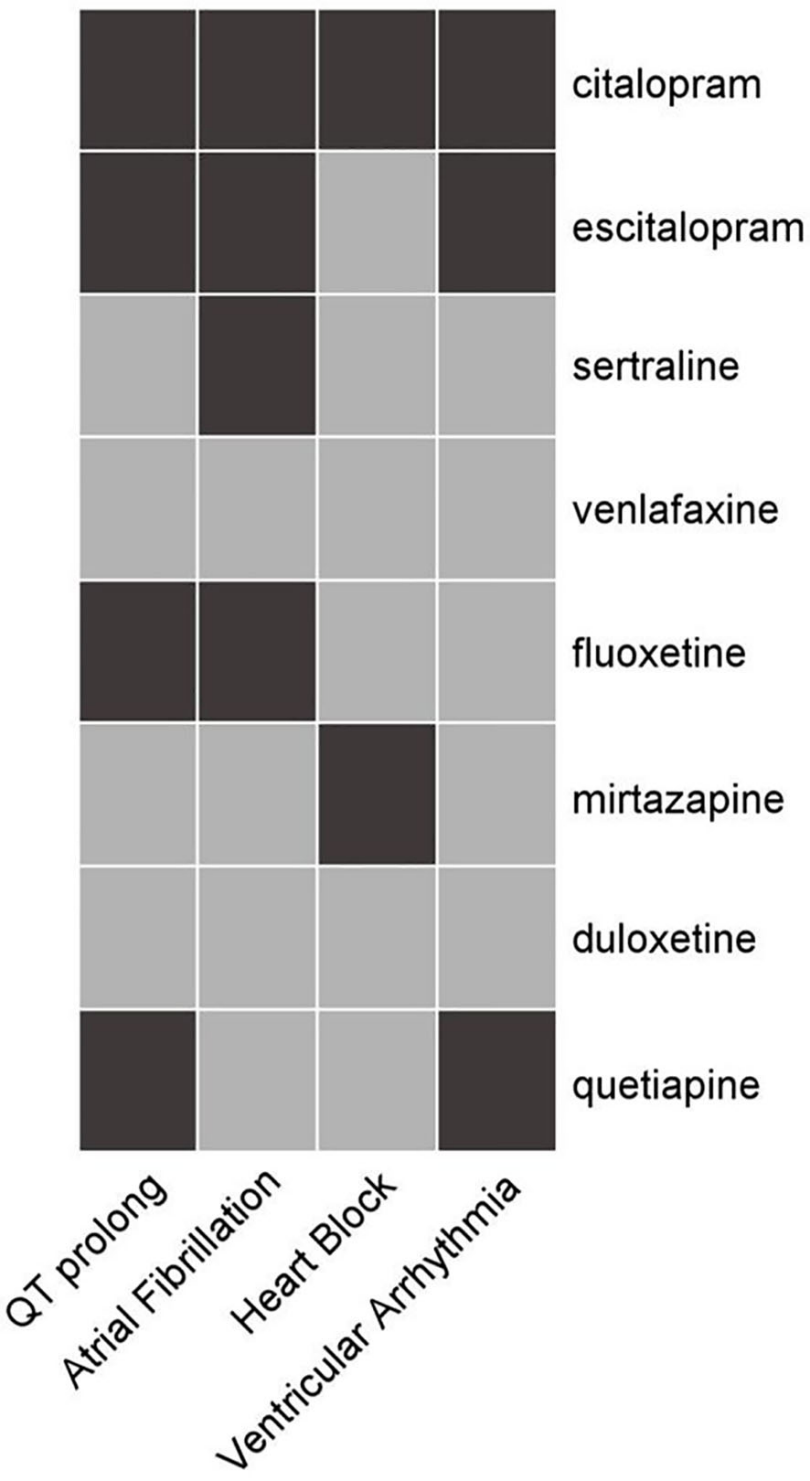
Heat map of correlations among arrhythmic outcomes and anti-depressants. Medications, listed on the right, include citalopram, escitalopram, sertraline, venlafaxine, fluoxetine, mirtazapine, duloxetine, and quetiapine. The cardiac conditions at the bottom are QT prolongation, atrial fibrillation, heart block, and ventricular arrhythmia. Black color indicates a significant pertinence between the drug and the AE, while gray color indicates no pertinence.

**Figure 1** shows the PV analysis of four arrhythmic endpoint events for eight antidepressants, accompanied by detailed statistical data in **Table 2**.

**Table 2:** Detection of pharmacovigilance (PV) of anti-depressive agents on 4 arrhythmia events (shown as PRR, ROR, IC and 95%confidence intervals).

QT prolongation/TdP	a	b	c	d	PRR(95% CI)	χ^2^	ROR(95% CI)	IC(95% CI)
Citalopram	381	2290	49824	694012	**2.31 (2.07, 2.57)**	241.669	**2.32 (2.08, 2.58)**	**1.08 (0.91, 1.21)**
Escitalopram	299	2372	50299	693537	1.73 (1.54, 1.95)	82.044	**1.74 (1.54, 1.96)**	**0.72 (0.53, 0.86)**
Sertraline	272	2399	81996	661840	0.92 (0.81, 1.04)	1.830	0.92 (0.81, 1.04)	-0.11 (-0.31, 0.03)
Venlafaxine	250	2421	78129	665707	0.88 (0.77, 1.00)	1.418	0.88 (0.77, 1.00)	-0.17 (-0.37, -0.01)
Fluoxetine	265	2406	50427	693409	1.51 (1.33, 1.72)	41.018	**1.51 (1.33, 1.72)**	**0.55 (0.34, 0.69)**
Mirtazapine	136	2535	35629	708207	1.07 (0.90, 1.27)	0.467	1.07 (0.90, 1.27)	0.09 (-0.20, 0.29)
Duloxetine	60	2701	79205	664541	0.17 (0.14, 0.24)	207.354	0.19 (0.14, 0.24)	-2.28 (-2.71, -1.97)
Quetiapine	120	2551	22643	721193	1.50 (1.25, 1.80)	18.405	**1.50 (1.25, 1.80)**	**0.56 (0.25, 0.77)**
Atrial fibrillation								
Citalopram	80	609	50125	695693	1.82 (1.44, 2.30)	25.468	**1.82 (1.44, 2.30)**	**0.78 (0.41, 1.05)**
Escitalopram	61	628	50537	695281	1.34 (1.03, 1.74)	4.378	**1.34 (1.03, 1.74)**	0.38 (-0.04, 0.69)
Sertraline	97	592	82171	663647	1.32 (1.07, 1.64)	6.268	**1.32 (1.07, 1.64)**	**0.35 (0.01, 0.59)**
Venlafaxine	67	622	78312	667506	0.92 (0.71, 1.81)	0.362	0.92 (0.71, 1.18)	-0.11 (-0.52, 0.18)
Fluoxetine	75	614	50617	695201	1.68 (1.32, 2.13)	17.627	**1.68 (1.32, 2.13)**	**0.68 (0.29, 0.95)**
Mirtazapine	25	664	35740	710078	0.75 (0.50, 1.12)	1.796	0.75 (0.50, 1.12)	-0.39 (-1.06, 0.08)
Duloxetine	78	611	79187	666631	1.08 (0.85, 1.36)	0.288	1.08 (0.85, 1.36)	0.09 (-0.28, 0.36)
Quetiapine	8	681	22755	723063	0.37 (0.19, 0.75)	7.69	0.37 (0.19, 0.75)	-1.34 (-2.55, -0.54)
Heart block								
Citalopram	59	596	50146	695706	1.37 (1.05, 1.79)	5.086	**1.37 (1.05, 1.80)**	0.42 (-0.01, 0.73)
Escitalopram	48	607	50550	695302	1.09 (0.81, 1.46)	0.233	1.09 (0.81, 1.46)	0.11 (-0.37, 0.45)
Sertraline	85	570	82183	663669	1.20 (0.96, 1.51)	2.364	1.20 (0.96, 1.51)	0.23 (-0.13, 0.49)
Venlafaxine	52	603	78327	667525	0.74 (0.55, 0.98)	4.305	0.73 (0.55, 0.98)	-0.40 (-0.86, -0.07)
Fluoxetine	43	612	50649	695203	0.96 (0.71, 1.31)	0.023	0.96 (0.71, 1.31)	-0.05 (-0.56, 0.31)
Mirtazapine	43	612	35722	710130	1.40 (1.03, 1.90)	4.142	**1.40 (1.03, 1.90)**	0.45 (-0.06, 0.81)
Duloxetine	20	635	79245	666607	0.27 (0.17, 0.41)	38.734	0.26 (0.17, 0.41)	-1.77 (-2.52, -1.25)
Quetiapine	28	627	22735	723117	1.42 (0.97, 2.07)	2.929	1.42 (0.97, 2.07)	0.48 (-0.15, 0.92)
Ventricular arrhythmia								
Citalopram	62	554	50150	695741	1.55 (1.19, 2.02)	10.427	**1.55 (1.19, 2.02)**	**0.58 (0.15, 0.88)**
Escitalopram	61	555	50537	695354	1.51 (1.16, 1.97)	9.038	**1.51 (1.16, 1.97)**	**0.54 (0.12, 0.85)**
Sertraline	65	551	82203	663688	0.95 (0.74, 1.23)	13.787	0.95 (0.74, 1.23)	-0.06 (-0.47, 0.23)
Venlafaxine	71	545	78308	667583	1.11 (0.87, 1.42)	0.586	1.11 (0.87, 1.42)	0.13 (-0.26, 0.42)
Fluoxetine	42	574	50650	695241	1.01 (0.73, 1.37)	0.003	1.01 (0.73, 1.37)	-0.01 (-0.51, 0.37)
Mirtazapine	25	591	35740	710151	0.84 (0.56, 1.25)	0.573	0.84 (0.56, 1.25)	-0.24 (-0.90, 0.24)
Duloxetine	18	598	79247	666644	0.25 (0.16, 0.41)	37.668	0.25 (0.16, 0.40)	-1.83 (-2.63, -1.28)
Quetiapine	43	573	22720	723171	**2.39 (1.75, 3.25)**	30.912	**2.39 (1.75, 3.26)**	**1.17 (0.67, 1.54)**

a, The number of reports of the drug of interest with the adverse event of interest; b, The number of reports of all other drugs with the adverse event of interest; c, The number of reports of the drug of interest with all other adverse events; d, The number of reports of all other drugs with all other adverse events; PRR, proportional reporting ratio; ROR, reporting odds ratio; IC, information component; CI, confidence interval.

Bolded values: indicate results with pharmacovigilance (PV) signals.

For QT prolongation/TdP, citalopram [PRR = 2.31, 95% confidence interval (CI): 2.07 to 2.57, *a* = 381, χ^2^ = 241.669; ROR = 2.32, 95% CI: 2.08 to 2.58; IC = 1.08, 95% CI: 0.91 to 1.21], escitalopram (ROR = 1.74, 95% CI: 1.54 to 1.96; IC = 0.72, 95% CI: 0.53 to 0.86), fluoxetine (ROR = 1.51, 95% CI: 1.33 to 1.72; IC = 0.55, 95% CI: 0.34 to 0.69), and quetiapine (ROR = 1.50, 95% CI: 1.25 to 1.80; IC = 0.56, 95% CI: 0.25 to 0.77) showed PV.

For AF, PV was detected for four drugs: citalopram (ROR = 1.82, 95% CI: 1.44 to 2.30; IC = 0.78, 95% CI: 0.41 to 1.05), escitalopram (ROR = 1.34, 95% CI: 1.03 to 1.74), sertraline (ROR = 1.32, 95% CI: 1.07 to 1.64; IC = 0.35, 95% CI: 0.01 to 0.59), and fluoxetine (ROR = 1.68, 95% CI: 1.32 to 2.13; IC = 0.68, 95% CI: 0.29 to 0.95).

For heart block, only two antidepressants, citalopram (ROR = 1.37, 95% CI: 1.05 to 1.80) and mirtazapine (ROR = 1.40, 95% CI: 1.03 to 1.90), prompted PV.

For ventricular arrhythmia, PV was detected in citalopram (ROR = 1.55, 95% CI: 1.19 to 2.02; IC = 0.58, 95% CI: 0.15 to 0.88), escitalopram (ROR = 1.51, 95% CI: 1.16 to 1.97; IC = 0.54, 95% CI: 0.12 to 0.85), and quetiapine (PRR = 2.39, 95% CI: 1.75 to 3.25, *a* = 43, χ^2^ = 30.912; ROR = 2.39, 95% CI: 1.75 to 3.26; IC = 1.17, 95% CI: 0.67 to 1.54).

### Constituent ratio of adverse events induced by drugs

For QT prolongation/TdP, citalopram demonstrated significantly higher constituent ratio compared with all other antidepressants analyzed: escitalopram [odds ratio (OR) = 1.29, 95% CI: 1.11 to 1.50], sertraline (OR = 2.31, 95% CI: 1.97 to 2.69), venlafaxine (OR = 2.39, 95% CI: 2.04 to 2.80), fluoxetine (OR = 1.46, 95% CI: 1.24 to 1.70), mirtazapine (OR = 2.00, 95% CI: 1.65 to 2.44), and quetiapine (OR = 1.44, 95% CI: 1.18 to 1.77). The most pronounced difference was observed compared with duloxetine (OR = 10.10, 95% CI: 7.69 to 13.26). Furthermore, escitalopram was associated with higher constituent ratio relative to several agents, including duloxetine (OR = 7.85, 95% CI: 5.95 to 10.36), sertraline (OR = 1.79, 95% CI: 1.52 to 2.11), and venlafaxine (OR = 1.86, 95% CI: 1.57 to 2.20). Conversely, duloxetine exhibited significantly lower constituent ratio compared to all other agents studied.

For AF, citalopram demonstrated higher constituent ratio compared with several agents: sertraline (OR = 1.35, 95% CI: 1.01 to 1.82), venlafaxine (OR = 1.87, 95% CI: 1.35 to 2.58), mirtazapine (OR = 2.28, 95% CI: 1.46 to 3.58), duloxetine (OR = 1.62, 95% CI: 1.19 to 2.21), and particularly quetiapine (OR = 4.54, 95% CI: 2.19 to 9.39). Quetiapine was associated with a markedly lower constituent ratio compared with all other studied antidepressants except mirtazapine. Moreover, mirtazapine showed significantly lower constituent ratio relative to all SSRIs in the analysis.

For heart block, duloxetine exhibited consistently lower constituent ratio relative to all comparator agents. A similar pattern was observed for venlafaxine. Notably, a substantial difference existed between SNRIs: venlafaxine was associated with a 2.63-fold higher constituent ratio than duloxetine (OR = 2.63; 95% CI: 1.57 to 4.41).

For ventricular arrhythmias, citalopram demonstrated significantly higher constituent ratio compared with sertraline (OR = 1.56, 95% CI: 1.10 to 2.22), fluoxetine (OR = 1.49, 95% CI: 1.01 to 2.21), mirtazapine (OR = 1.77, 95% CI: 1.11 to 2.81), and most pronouncedly duloxetine (OR = 5.44, 95% CI: 3.22 to 9.20). Duloxetine exhibited significantly lower constituent ratio compared with all comparator antidepressants. Conversely, quetiapine was associated with a substantially higher constituent ratio relative to all other studied agents.

**Figure 2:**
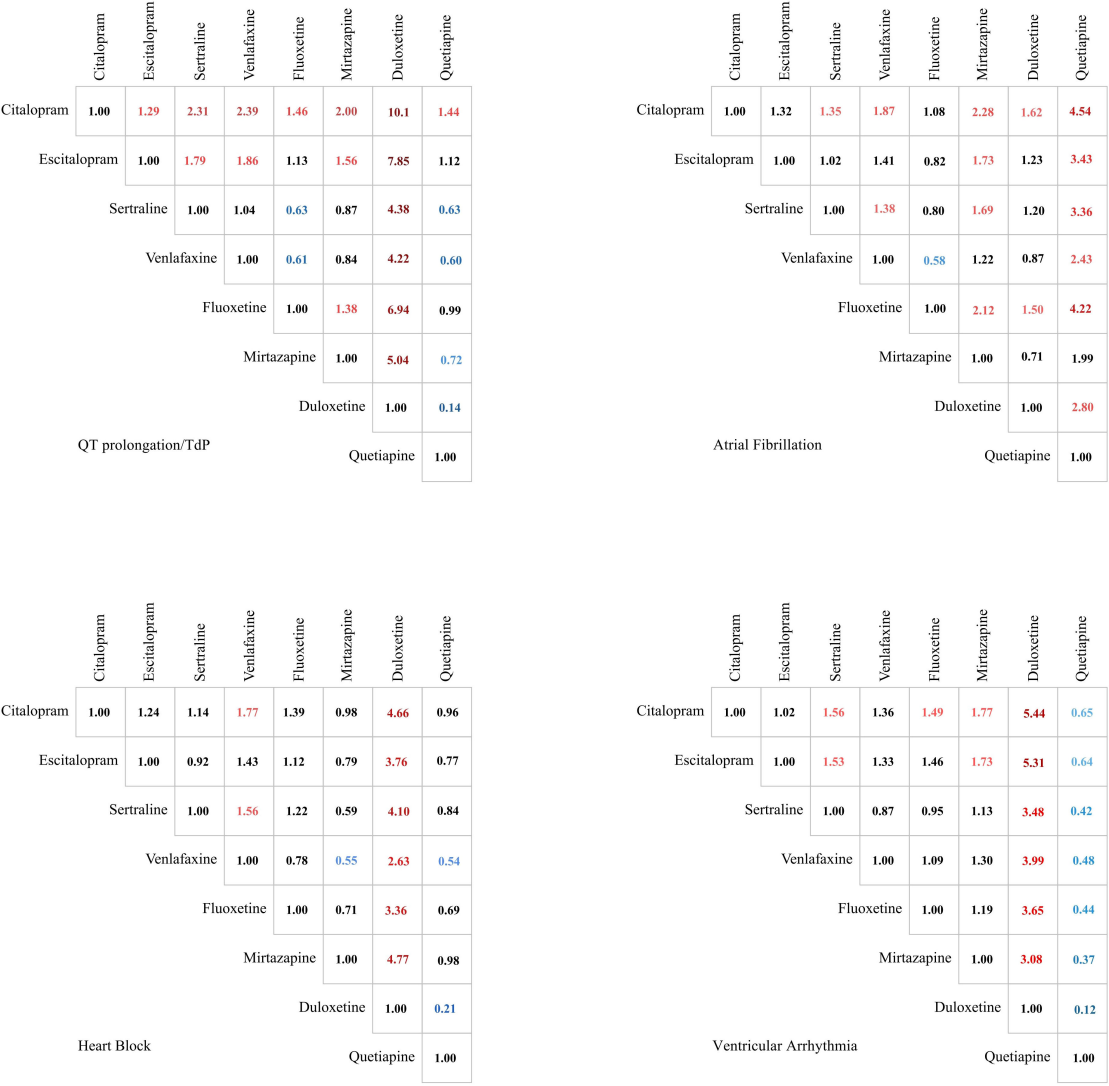
Comparison of the constituent ratios of 4 Arrhythmia events caused by drugs in treating depression. Significant results are color-coded. Values greater than 1 are marked in red, while values less than 1 are indicated in blue. The color intensity reflects the magnitude of deviation from the baseline value of 1.

The specific signal value is displayed in **Figure 2** and **Supplementary Table 6**.

### Severity of reaction outcomes for patients with drug-related arrhythmic events

For QT prolongation/TdP, citalopram demonstrated significantly lower odds of severe events (potential progression to fatal outcomes) than sertraline (OR = 0.54, 95% CI: 0.37 to 0.79), venlafaxine (OR = 0.49, 95% CI: 0.34 to 0.72), and quetiapine (OR = 0.47, 95% CI: 0.30 to 0.76). Escitalopram maintained a similarly favorable safety profile. In contrast, venlafaxine showed significantly increased odds of serious outcomes, as did fluoxetine (OR = 1.74, 95% CI: 1.13 to 2.69) and duloxetine (OR = 2.20, 95% CI: 1.02 to 4.75).

For AF, significant odds differences primarily emerged in comparisons against fluoxetine. Specifically, escitalopram (OR = 2.84, 95% CI: 1.25 to 6.44), sertraline (OR = 2.26, 95% CI: 1.04 to 4.92), venlafaxine (OR = 3.83, 95% CI: 1.61 to 9.12), and duloxetine (OR = 0.35, 95% CI: 0.16 to 0.78; duloxetine as reference) demonstrated significantly higher odds of severe AF compared with fluoxetine. No other pairwise comparisons reached statistical significance for this endpoint.

For heart block, citalopram demonstrated significantly higher odds of severe events compared with most comparators: escitalopram (OR = 4.35, 95% CI: 1.83 to 10.33), sertraline (OR = 2.39, 95% CI: 1.13 to 5.05), venlafaxine (OR = 22.97, 95% CI: 6.03 to 87.60), mirtazapine (OR = 7.19, 95% CI: 2.38 to 21.68), and duloxetine (OR = 10.69, 95% CI: 1.13 to 101.32). Fluoxetine showed comparable odds to citalopram. Conversely, venlafaxine exhibited significantly lower odds than all other antidepressants except mirtazapine and duloxetine, where odds were statistically indistinguishable.

For ventricular arrhythmia, escitalopram demonstrated significantly lower odds of severe events compared with multiple comparators: citalopram (OR = 3.88, 95% CI: 1.68 to 8.95; escitalopram as reference), sertraline (OR = 0.37, 95% CI: 0.16 to 0.86), venlafaxine (OR = 0.26, 95% CI: 0.12 to 0.59), fluoxetine (OR = 0.27, 95% CI: 0.10 to 0.75), and mirtazapine (OR = 0.17, 95% CI: 0.06 to 0.53). Conversely, quetiapine showed significantly higher odds than all antidepressants studied, though its odds did not statistically differ from mirtazapine or duloxetine.

**Figure 3:**
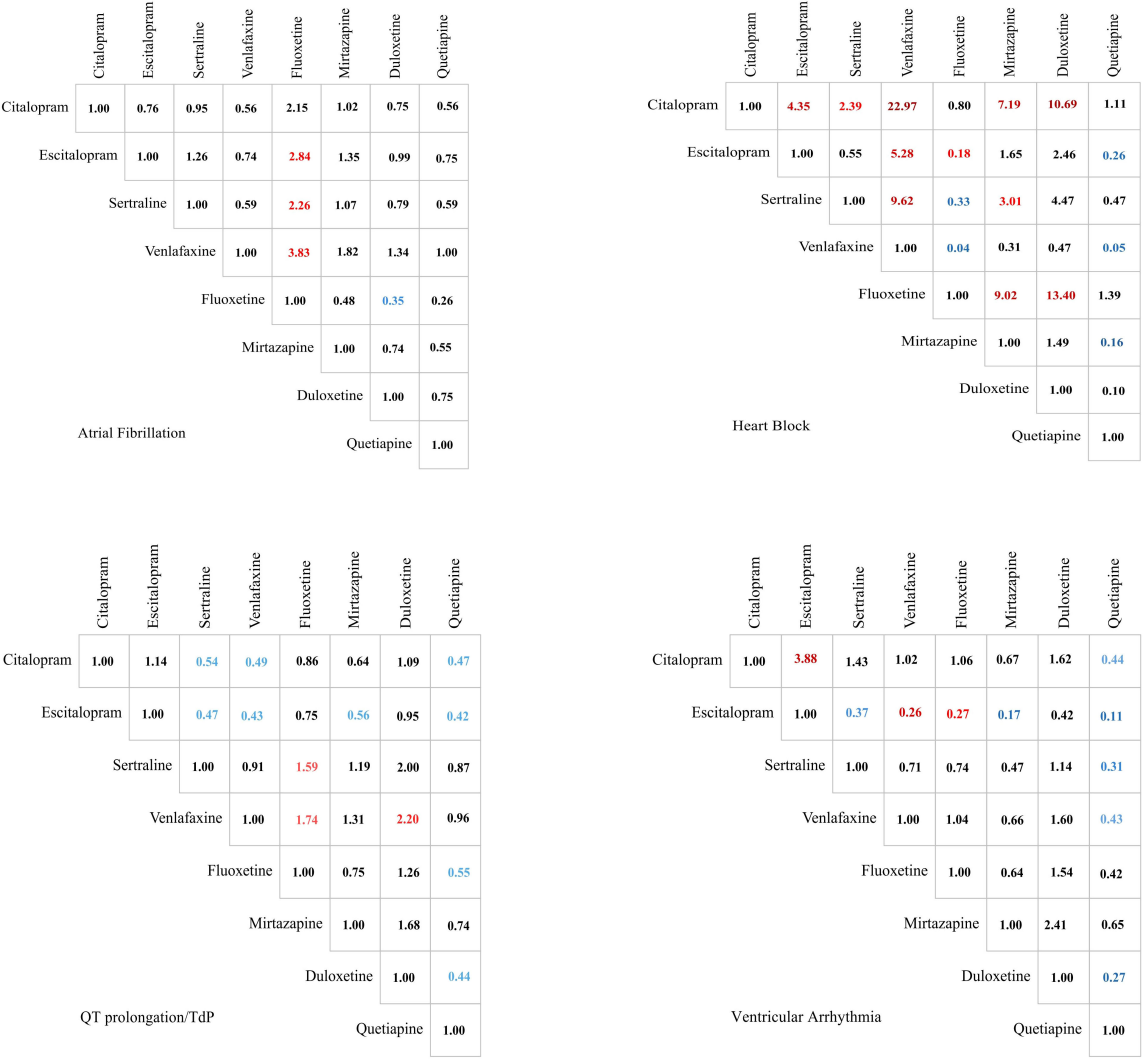
Comparison of the serious degree of adverse events' severities of 4 arrhythmia events caused by drugs in treating depression. Significant results are color-coded. Values greater than 1 are marked in red, while values less than 1 are indicated in blue. The color intensity reflects the magnitude of deviation from the baseline value of 1.

The specific signal value is displayed in **Figure 3** and **Supplementary Table 7**.

## Discussion

To the best of our knowledge, this is the first real-world study investigating the PV of antidepressants on arrhythmia in treating depression deploying Bayesian disproportional analysis.

Descriptive analyses indicated that middle-aged and older adults and women were more prone to developing drug-related arrhythmia, which was consistent with the observations made by Chen et al. (9).

Previous investigations have characterized a “biphasic effect” of estrogen on inflammatory responses, wherein low estrogen levels exert a pro-inflammatory effect, whereas high levels attenuate inflammation through anti-inflammatory mechanisms (10, 11). Mechanistically, inflammatory mediators impair tryptophan hydroxylase activity, thereby limiting serotonin (5-HT) biosynthesis. Notably, sustained inflammatory states further induce hippocampal glucocorticoid receptor (GR) resistance, promoting the dysregulated HPA axis with consequent hypercortisolemia—a pathogenic contributor to depression development (12). Collectively, these findings indicate a heightened neuroendocrine vulnerability to depression in female relative to male patients, potentially attributable to cyclical sex hormone fluctuations (13).

A number of analyses indicated that sexual hormones and their effects on HPA axis activity had a combined effect on the onset and prognosis of depression. It was noteworthy that these effects displayed notable differences when observed in groups of women at different reproductive stages (14). Furthermore, the high prevalence of depression and cardiovascular risk factors such as hypertension and metabolic syndrome in peri- and post-menopausal women, when baseline estrogen levels decrease, might contribute in a synergistic manner to the observed gender differences in drug-related arrhythmic events.

With regard to QT prolongation, citalopram, escitalopram, fluoxetine, and quetiapine demonstrated PV, while citalopram exhibited the most intensive signal, numerically; moreover, sertraline exerted no PV. This finding was in partial congruity with the results of a meta-analysis conducted by Beach et al. (3), which showed significant PV signal of sertraline in addition to the four aforementioned agents on this endpoint. However, our findings were consistent with those of Glassman et al. (15), in which a PV signal associating sertraline with QT interval prolongation was detected.

It was widely acknowledged that the pathological mechanism by which SSRIs induce QT prolongation is the blockade of the main component of cardiomyocyte repolarization, *I*_Kr_, the fast delayed rectifier potassium current, which acted synergistically with *I*_Ks_ in the repolarization process. The human ether-a-go-go related gene (hERG) regulated *I*_Kr_. The Kv11.1 protein encoded by hERG constitutes the α-subunit of the potassium channel protein. Consequently, aberrant expression of the hERG gene or pharmacological blockade of the hERG potassium channel could result in slowed potassium efflux, prolonged repolarization, and, consequently, a prolonged QT interval. At the cellular level, Witchel et al. (16) demonstrated a dose-dependent inhibitory effect of citalopram on hERG.

The relationship between quetiapine and QT prolongation has not been clearly established in previous studies. A study that employed concentration-QT modeling and simulation to assess the QT prolongation effect of quetiapine extended-release (XR) formulations found that quetiapine at therapeutic doses was not clinically correlated with QT prolongation (17), which is contrary to our findings. Notably, the analysis conducted by Fukushi et al. (17) included five studies in which confounding factors might be present. Additionally, the use of a small sample might have compromised the results of that study.

By investigating the constituent ratio of QT prolongation/TdP, the following ranking according to OR has been made: citalopram > escitalopram = fluoxetine = quetiapine. A search of the literature did not yield any analogous study that could be used to support this conclusion; in other words, this finding was unprecedented. These findings highlight the need for clinicians to exercise greater caution in citalopram selection during clinical practice to mitigate the risk of drug-induced QT prolongation/TdP. In terms of degree of seriousness, it appeared that QT prolongation/TdP due to quetiapine had a severer prognosis, which was in agreement with the findings by Wang et al. (18), who found that severe QT prolongation (SQTP) in quetiapine users was significantly associated with ventricular arrhythmia (OR = 2.84, 95% CI: 1.95 to 4.13) and sudden cardiac death (OR = 2.29, 95% CI: 1.44 to 3.66).

Although the incidence of QT prolongation/TdP was significantly higher with escitalopram compared to other drugs, the risk of serious outcomes was low. Citalopram exhibited similar results. It was hypothesized that this might be attributed to the effect of the inward current *I*_Ca,L_. It was established that TdP is closely associated with the phenomenon of early after-depolarization (EAD), which is a process whereby the cell membrane undergoes a second depolarization during the action potential (AP) due to an increase in inward current and/or a decrease in outward current (19). In addition to the blockade of hERG channels, which results in *I*_Kr_ inhibition, another mechanism that contributes to the development of EAD is the abnormal depolarization of the cell membrane caused by an increase in the inward flow of L-type calcium ions (20). EAD is situated within long action potential duration (APD) islands, facilitating a dynamic increase in the steep voltage gradient (VG). VG at the APD island boundary activates L-type calcium currents in circumscribed regions of the myocardium, thereby electrically stimulating the PVC to trigger TdP (21). Escitalopram and citalopram might result in a reduction in calcium flux through direct action on *I*_Ca,L_, which might counteract the QT prolongation associated with *I*_hERG_ or may minimize EAD production and thus reduce malignant arrhythmic events (16).

The majority of clinical studies that have examined the electrical activity of the atrial myocardium have focused on AF. In this regard, citalopram, escitalopram, sertraline, and fluoxetine exhibited robust evidence of adverse effects. A meta-analysis conducted by Cao et al. (22) substantiated that utilization of antidepressants remarkably elevated the likelihood of AF (RR = 1.37, 95% CI: 1.16 to 1.61). The currently proposed mechanism might be ascribed to the fact that serotoninergic antidepressants increased the risk of AF by acting on the 5-HT4 receptor, increasing intracellular calcium ion concentrations, and increasing the amplitude of the pacing current in atrial myocytes (23). With regard to the constituent ratios, the only positive result was a significantly increased risk of AF with citalopram compared to sertraline. In addition, escitalopram, sertraline, venlafaxine, and duloxetine all resulted in a worse prognosis of AF compared to fluoxetine in our study.

With regard to heart block, citalopram and mirtazapine demonstrated a notable PV. Previous case reports have indicated that citalopram may increase the risk of bradycardia and heart block in elderly patients (24). However, there is a paucity of clinical studies that have been able to confirm this correlation, and our study contributes to this gap in the literature. It is hypothesized that citalopram inhibits L-type calcium channel currents, which may lead to impaired atrioventricular conduction and the induction of prolongation of the P–R interval and atrioventricular block (25).

Mirtazapine belongs to NaSSAs (noradrenergic and specific serotonergic antidepressants), which has a unique pharmacological profile, including potent antagonism of central alpha 2-adrenergic autoreceptors and heteroreceptors and antagonism of both serotonin 5-hydroxytryptamine-2 (5-HT2) and 5-HT3 receptors (26). Nevertheless, a descriptive study of adverse cardiovascular events based on the German National Continuous Pharmacovigilance Programme (AMSP) found that mirtazapine was associated with a significantly lower risk of cardiac arrhythmia compared to other antidepressants (6%, *p* < 0.001). However, the limited number of cases may have contributed to biased results (27).

Although fluoxetine was not identified as an alert drug for heart block and the constituent ratio was not significantly increased in comparison to other drugs, the prognosis was similar to that of citalopram. This highlighted the necessity for clinicians to pay closer attention to fluoxetine-induced heart block, avoiding the evolution into malignant arrhythmia. It was noteworthy that the SNRIs demonstrated a notable reduction in constituent ratio and prognostic severity in comparison to numerous other drugs that provided indirect evidence supporting the safety profile of this pharmacological class.

Citalopram, escitalopram, and quetiapine showed numerical PV of ventricular arrhythmia. The conclusions of previous studies regarding the associations between ventricular arrhythmia and escitalopram as well as citalopram were inconsistent. A nationwide nested case–control study in Denmark demonstrated that the risk of out-of-hospital cardiac arrest was found to be increased in patients taking high-dose citalopram (>20 mg, HR = 1.46, 95% CI: 1.16 to 1.75) and high-dose escitalopram (>10 mg, HR = 1.43, 95% CI: 1.16 to 1.75) (28). However, a study by Aakjaer et al. (29), which defined ventricular tachycardia, ventricular fibrillation, ventricular flutter, ventricular pre-systole, and cardiac arrest as serious arrhythmia, indicated that neither citalopram (RR = 0.87, 95% CI: 0.62 to 1.22) nor escitalopram (RR = 0.86, 95% CI: 0.53 to 1.40) significantly increased the risk of serious arrhythmia.

Wu et al. (30) indicated a strong association between quetiapine and VA/SCD (OR = 1.29, 95% CI: 1.07 to 1.56), which aligned with our conclusions. The potential mechanism may be attributed to the blocking effect of quetiapine on hERG potassium channels, while Wu observed that the strength of this blocking effect is proportional to the risk of SCD/VA.

No significant difference was observed between citalopram and escitalopram in terms of the constituent ratio. However, the incidence of ventricular arrhythmia was found to be significantly higher with citalopram compared to sertraline. A retrospective study conducted in Canada corroborated our findings, confirming that citalopram was associated with a higher risk of ventricular arrhythmias compared to paroxetine or sertraline (RR = 1.53, 95% CI: 1.03 to 2.29) (31). Our findings diverge slightly in that the risk of ventricular arrhythmia was not markedly elevated with escitalopram (RR = 0.84, 95% CI: 0.42 to 1.68) in Qirjazi et al.’s (31) study, which suggested that some endpoint events might have been overlooked due to the low incidence of VA (0.05%) observed over a relatively short follow-up period of 90 days.

In terms of prognostic severity, the ranking is as follows: quetiapine > citalopram > escitalopram. The precise mechanisms underlying this phenomenon remained unclear. One study identified a slightly greater effect of escitalopram on cardiac AP triangulation and prolonged complete repolarization. However, a 10-fold concentration of escitalopram reduced the time to early repolarization (32), which may indicate that the R(−)-enantiomer plays a specific role in cardiotoxicity compared with the S(+)-enantiomer. It was also noteworthy that quetiapine showed a significantly increased constituent ratio and a significantly higher prognostic risk. However, there is no relevant literature to compare with our findings, which warrants further research exploration.

Notably, in addition to focusing on arrhythmia caused by antidepressants, psychiatrists must also comprehensively assess the cardiac condition of patients before prescribing and exclude potential arrhythmia risk factors such as electrolyte disorders, thyroid dysfunction, underlying cardiovascular disease, or concomitant use of multiple medications. It is incumbent upon cardiovascular physicians to conduct regular follow-up and monitoring of cardiac function and electrocardiograms in patients who are on arrhythmic PV medications. Furthermore, they must exercise individualized selection of antidepressants with preexisting cardiovascular underlying diseases. Upon the occurrence of an arrhythmia, the administration of the pharmaceutical agent in question should be terminated immediately, and safer alternatives to SNRIs may be available. Thereafter, efforts should be undertaken to restore the rhythm while maintaining electrolyte balance. In the event that these efforts prove unsuccessful, electric defibrillation can be considered.

## Limitation

First, reliance on the FAERS database, which collects spontaneous, voluntary AE reports, introduces potential limitations including data incompleteness, duplication, and inaccuracies. This complicates direct comparisons and reliable estimation of true risk for specific drug–event associations.

Second, the absence of detailed clinical data, such as baseline patient characteristics, dosing regimens, and treatment duration, limits the control of confounding factors and impedes causal inference.

Third, the analysis was restricted to eight medications, excluding TCAs, other atypical antidepressants, and other SGAs, potentially limiting the generalizability of the findings. This will constitute a major focus of our future research.

Fourth, differential reporting rates may bias results, as more frequently prescribed drugs inherently generate more reports, potentially distorting PRR.

Fifth, underreporting of arrhythmias may occur, as their detection often requires prolonged monitoring beyond typical PV windows, leading to potential reporting bias where some incident cases remain undocumented.

## Conclusion

Citalopram and escitalopram (classified as SSRIs) exhibited the strongest correlations with arrhythmic occurrences. Quetiapine (classified as an SGA) demonstrated higher risk and worse prognosis on QT prolongation/TdP and ventricular arrhythmic events. Venlafaxine and duloxetine (classified as SNRIs) did not show any PV of any arrhythmia and had lower risks and a lower degree of AEs. Certainly, more head-to-head related studies are warranted.

## Data Availability

Publicly available datasets were analyzed in this study. This data can be found here: https://www.fda.gov/drugs/drug-approvals-and-databases/fda-adverse-event-reporting-system-faers-database.
