# Supplementary material for: Arrhythmic events pertinent with antidepressants: a Bayesian disproportional analysis mining the FDA Adverse Event Reporting System database
Source: Front Psychiatry. 2025 Sep 29;16:1637471. doi: 10.3389/fpsyt.2025.1637471 (PMC12515912; doi:10.3389/fpsyt.2025.1637471)
Supplement: Supplementary file 1 [file Table1.pdf]

Table 1. Cardiovascular adverse events as 8 narrow entities according to MedDRA 27.0.

SMQ code	SMQ name
20000001	QT prolongation/Torsade de pointes
10003658	Atrial fibrillation
10019252	heart block
10047281	Ventricular arrhythmia
