# Supplementary material for: Arrhythmic events pertinent with antidepressants: a Bayesian disproportional analysis mining the FDA Adverse Event Reporting System database
Source: Front Psychiatry. 2025 Sep 29;16:1637471. doi: 10.3389/fpsyt.2025.1637471 (PMC12515912; doi:10.3389/fpsyt.2025.1637471)
Supplement: Supplementary file 2 [file Table2.pdf]

Table 2. Overview of QT prolongation/Torsade de pointes According to the Medical Dictionary for Regulatory Activities (MedDRA) Preferred Terms.

Preferred Term	Study Group
Long QT syndrome	QT prolongation/Torsade de pointes
Electrocardiogram QT prolonged	QT prolongation/Torsade de pointes
Torsade de pointes	QT prolongation/Torsade de pointes
