# Supplementary material for: Arrhythmic events pertinent with antidepressants: a Bayesian disproportional analysis mining the FDA Adverse Event Reporting System database
Source: Front Psychiatry. 2025 Sep 29;16:1637471. doi: 10.3389/fpsyt.2025.1637471 (PMC12515912; doi:10.3389/fpsyt.2025.1637471)
Supplement: Supplementary file 3 [file Table3.pdf]

Table 3. Overview of Heart Block According to the Medical Dictionary for Regulatory Activities (MedDRA) Preferred Terms.

Preferred Term	Study Group
Atrioventricular block	Heart block
Atrioventricular block first degree	Heart block
Atrioventricular block second degree	Heart block
Atrioventricular block complete	Heart block
Bifascicular block	Heart block
Bundle branch block	Heart block
Bundle branch block left	Heart block
Bundle branch block right	Heart block
Sinoatrial block	Heart block
