# Supplementary material for: Arrhythmic events pertinent with antidepressants: a Bayesian disproportional analysis mining the FDA Adverse Event Reporting System database
Source: Front Psychiatry. 2025 Sep 29;16:1637471. doi: 10.3389/fpsyt.2025.1637471 (PMC12515912; doi:10.3389/fpsyt.2025.1637471)
Supplement: Supplementary file 4 [file Table4.pdf]

Table 4. Overview of Ventricular Arrhythmia According to the Medical Dictionary for Regulatory Activities (MedDRA) Preferred Terms.

Preferred Term	Study Group
Ventricular arrhythmia	ventricular arrhythmia
Ventricular tachyarrhythmia	ventricular arrhythmia
Ventricular tachycardia	ventricular arrhythmia
Ventricular extrasystoles	ventricular arrhythmia
Ventricular fibrillation	ventricular arrhythmia
Ventricular flutter	ventricular arrhythmia
Ventricular asystole	ventricular arrhythmia
