# Supplementary material for: Arrhythmic events pertinent with antidepressants: a Bayesian disproportional analysis mining the FDA Adverse Event Reporting System database
Source: Front Psychiatry. 2025 Sep 29;16:1637471. doi: 10.3389/fpsyt.2025.1637471 (PMC12515912; doi:10.3389/fpsyt.2025.1637471)
Supplement: Supplementary file 5 [file Table5.pdf]

Table 5. ROR, PRR and BCPNN methods, formulas, and thresholds.

Algorithms	Equation	Criteria
PRR	$\text{PRR} = \frac{a/(a+c)}{b/(b+d)}$<br>$\text{PRR95\% CI} = e^{\text{In(PRR)}\pm 1.96\sqrt{\frac{1}{a}-\frac{1}{a+c}+\frac{1}{b}-\frac{1}{b+d}}}$<br>$\chi^2_{\text{yates}} = \frac{(a+b+c+d)\times(\lvert a\times d\rvert-\lvert b\times c\rvert-(a+b+c+d)/2)^2}{(a+c)\times(a+b)\times(b+d)\times(d+c)}$	The number of cases $\geq$ 3, PRR $\geq$ 2 and $\chi2$ analysis ($\chi2$) $\geq$ 4
ROR	$\text{ROR}=\frac{(a/c)}{(b/d)}=\frac{ad}{bc}$<br>$\text{ROR95\% CI}=e^{\text{In(ROR)}\pm 1.96\sqrt{\frac{1}{a}+\frac{1}{b}+\frac{1}{c}+\frac{1}{d}}}$	The lower limit of 95% confidence intervals (CI) of ROR is > 1
BCPNN	$\text{IC} = \log_2 \frac{a+0.5}{a_{\exp}+0.5}$<br>$a_{\exp}=\frac{(a+b)\times(a+c)}{(a+b+c+d)}$<br>$\text{IC}_{025}=\text{IC-3.3}\times(a+0.5)^{-1/2}\text{-2}\times(a+0.5)^{-3/2}$	The IC025 > 0

In the above equation, a = The number of reports of the drug of interest with the adverse event of interest. b = The number of

reports of all other drugs with the adverse event of interest. c = The number of reports of the drug of interest with all other adverse events. d = The number of reports of all other drugs with all other adverse events.
