# Supplementary material for: Arrhythmic events pertinent with antidepressants: a Bayesian disproportional analysis mining the FDA Adverse Event Reporting System database
Source: Front Psychiatry. 2025 Sep 29;16:1637471. doi: 10.3389/fpsyt.2025.1637471 (PMC12515912; doi:10.3389/fpsyt.2025.1637471)
Supplement: Supplementary file 6 [file Table6.pdf]

Table 6. Comparison of the Constituent Ratios of 4 Arrhythmia events Caused by Drugs in Treating depression.

QT prolongation/TdP	Pairwise comparison	odds ratios (ORs)
	Citalopram vs.Escitalopram	**1.29(1.11,1.50)**
	Citalopram vs. Sertraline	**2.31(1.97,2.69)**
	Citalopram vs. Venlafaxine	**2.39(2.04,2.80)**
	Citalopram vs. Fluoxetine	**1.46(1.24,1.70)**
	Citalopram vs. Mirtazapine	**2.00(1.65,2.44)**
	Citalopram vs. Duloxetine	**10.10(7.69,13.26)**
	Citalopram vs. Quetiapine	**1.44(1.18,1.77)**
	Escitalopram vs. Sertraline	**1.79(1.52,2.11)**
	Escitalopram vs. Venlafaxine	**1.86(1.57,2.20)**
	Escitalopram vs. Fluoxetine	1.13(0.96,1.34)
	Escitalopram vs. Mirtazapine	**1.56(1.27,1.91)**
	Escitalopram vs. Duloxetine	**7.85(5.95,10.36)**
	Escitalopram vs. Quetiapine	1.12(0.91,1.39)
	Sertraline vs. Venlafaxine	1.04(0.87,1.23)
	Sertraline vs. Fluoxetine	**0.63(0.53,0.75)**
	Sertraline vs. Mirtazapine	0.87(0.71,1.07)
	Sertraline vs. Duloxetine	**4.38(3.11,5.79)**
	Sertraline vs. Quetiapine	**0.63(0.51,0.78)**
	Venlafaxine vs. Fluoxetine	**0.61(0.51,0.72)**
	Venlafaxine vs. Mirtazapine	0.84(0.68,1.03)
	Venlafaxine vs. Duloxetine	**4.22(3.19,5.60)**
	Venlafaxine vs. Quetiapine	**0.60(0.49,0.75)**
	Fluoxetine vs. Mirtazapine	**1.38(1.12,1.69)**
	Fluoxetine vs. Duloxetine	**6.94(5.24,9.18)**

	Fluoxetine vs. Quetiapine	0.99(0.80,1.23)
	Mirtazapine vs. Duloxetine	**5.04(3.72,6.83)**
	Mirtazapine vs. Quetiapine	**0.72(0.56,0.92)**
	Duloxetine vs. Quetiapine	**0.14(0.11,0.20)**
Atrial Fibrillation	Citalopram vs.Escitalopram	1.32(0.95,1.85)
	Citalopram vs. Sertraline	**1.35(1.01,1.82)**
	Citalopram vs. Venlafaxine	**1.87(1.35,2.58)**
	Citalopram vs Fluoxetine	1.08(0.79,1.48)
	Citalopram vs. Mirtazapine	**2.28(1.46,3.58)**
	Citalopram vs. Duloxetine	**1.62(1.19,2.21)**
	Citalopram vs. Quetiapine	**4.54(2.19,9.39)**
	Escitalopram vs. Sertraline	1.02(0.74,1.41)
	Escitalopram vs. Venlafaxine	1.41(0.99,1.99)
	Escitalopram vs. Fluoxetine	0.82(0.58,1.14)
	Escitalopram vs. Mirtazapine	**1.73(1.08,2.75)**
	Escitalopram vs. Duloxetine	1.23(0.88,1.71)
	Escitalopram vs. Quetiapine	**3.43(1.64,7.18)**
	Sertraline vs. Venlafaxine	**1.38(1.01,1.88)**
	Sertraline vs. Fluoxetine	0.80(0.59,1.08)
	Sertraline vs. Mirtazapine	**1.69(1.09,2.62)**
	Sertraline vs. Duloxetine	1.20(0.89,1.62)
	Sertraline vs. Quetiapine	**3.36(1.63,6.91)**
	Venlafaxine vs. Fluoxetine	**0.58(0.42,0.80)**
	Venlafaxine vs. Mirtazapine	1.22(0.77,1.94)
	Venlafaxine vs. Duloxetine	0.87(0.63,1.20)
	Venlafaxine vs. Quetiapine	**2.43(1.17,5.07)**

	Fluoxetine vs. Mirtazapine	**2.12(1.35,3.33)**
	Fluoxetine vs. Duloxetine	**1.50(1.10,2.07)**
	Fluoxetine vs. Quetiapine	**4.22(2.03,8.74)**
	Mirtazapine vs. Duloxetine	0.71(0.45,1.11)
	Mirtazapine vs. Quetiapine	1.99(0.90,4.41)
	Duloxetine vs. Quetiapine	**2.80(1.35,5.80)**
Heart Block	Citalopram vs.Escitalopram	1.24(0.85,1.81)
	Citalopram vs. Sertraline	1.14(0.82,1.59)
	Citalopram vs. Venlafaxine	**1.77(1.22,2.57)**
	Citalopram vs Fluoxetine	1.39(0.94,2.05)
	Citalopram vs. Mirtazapine	0.98(0.66,1.45)
	Citalopram vs. Duloxetine	**4.66(2.81,7.74)**
	Citalopram vs. Quetiapine	0.96(0.61,1.50)
	Escitalopram vs. Sertraline	0.92(0.64,1.31)
	Escitalopram vs. Venlafaxine	1.43(0.97,2.12)
	Escitalopram vs. Fluoxetine	1.12(0.74,1.69)
	Escitalopram vs. Mirtazapine	0.79(0.52,1.19)
	Escitalopram vs. Duloxetine	**3.76(2.23,6.34)**
	Escitalopram vs. Quetiapine	0.77(0.48,1.23)
	Sertraline vs. Venlafaxine	**1.56(1.10,2.20)**
	Sertraline vs. Fluoxetine	1.22(0.84,1.76)
	Sertraline vs. Mirtazapine	0.59(0.60,1.24)
	Sertraline vs. Duloxetine	**4.10(2.52,6.67)**
	Sertraline vs. Quetiapine	0.84(0.55,1.29)
	Venlafaxine vs. Fluoxetine	0.78(0.52,1.17)
	Venlafaxine vs. Mirtazapine	**0.55(0.37,0.83)**

	Venlafaxine vs. Duloxetine	**2.63(1.57,4.41)**
	Venlafaxine vs. Quetiapine	**0.54(0.34,0.85)**
	Fluoxetine vs. Mirtazapine	0.71(0.46,1.08)
	Fluoxetine vs. Duloxetine	**3.36(1.98,5.72)**
	Fluoxetine vs. Quetiapine	0.69(0.43,1.11)
	Mirtazapine vs. Duloxetine	**4.77(2.81,8.11)**
	Mirtazapine vs. Quetiapine	0.98(0.61,1.57)
	Duloxetine vs. Quetiapine	**0.21(0.12,0.36)**
Ventricular Arrhythmia	Citalopram vs. Escitalopram	1.02(0.72,1.46)
	Citalopram vs. Sertraline	**1.56(1.10,2.22)**
	Citalopram vs. Venlafaxine	1.36(0.97,1.92)
	Citalopram vs. Fluoxetine	**1.49(1.01,2.21)**
	Citalopram vs. Mirtazapine	**1.77(1.11,2.81)**
	Citalopram vs. Duloxetine	**5.44(3.22,9.20)**
	Citalopram vs. Quetiapine	**0.65(0.44,0.96)**
	Escitalopram vs. Sertraline	**1.53(1.08,2.17)**
	Escitalopram vs. Venlafaxine	1.33(0.95,1.88)
	Escitalopram vs. Fluoxetine	1.46(0.98,2.16)
	Escitalopram vs. Mirtazapine	**1.73(1.08,2.75)**
	Escitalopram vs. Duloxetine	**5.31(3.14,8.99)**
	Escitalopram vs. Quetiapine	**0.64(0.43,0.94)**
	Sertraline vs. Venlafaxine	0.87(0.62,1.22)
	Sertraline vs. Fluoxetine	0.95(0.65,1.41)
	Sertraline vs. Mirtazapine	1.13(0.71,1.79)
	Sertraline vs. Duloxetine	**3.48(2.07,5.87)**
	Sertraline vs. Quetiapine	**0.42(0.28,0.61)**

Venlafaxine vs. Fluoxetine	1.09(0.75,1.60)
Venlafaxine vs. Mirtazapine	1.30(0.82,2.05)
Venlafaxine vs. Duloxetine	**3.99(2.38,6.70)**
Venlafaxine vs. Quetiapine	**0.48(0.33,0.70)**
Fluoxetine vs. Mirtazapine	1.19(0.72,1.95)
Fluoxetine vs. Duloxetine	**3.65(2.10,6.34)**
Fluoxetine vs. Quetiapine	**0.44(0.29,0.67)**
Mirtazapine vs. Duloxetine	**3.08(1.68,5.65)**
Mirtazapine vs. Quetiapine	**0.37(0.23,0.61)**
Duloxetine vs. Quetiapine	**0.12(0.07,0.21)**
