# Supplementary material for: Arrhythmic events pertinent with antidepressants: a Bayesian disproportional analysis mining the FDA Adverse Event Reporting System database
Source: Front Psychiatry. 2025 Sep 29;16:1637471. doi: 10.3389/fpsyt.2025.1637471 (PMC12515912; doi:10.3389/fpsyt.2025.1637471)
Supplement: Supplementary file 7 [file Table7.pdf]

Table 7. Comparison of the Serious Degree of Adverse Events' Severities of 4 Arrhythmia events Caused by Drugs in treating depression. (shown as odds ratios and 95% confidence intervals).

QT prolongation/TdP	Pairwise comparison	odds ratios (ORs)
	Citalopram vs.Escitalopram	1.14 (0.78, 1.68)
	Citalopram vs. Sertraline	**0.54 (0.37, 0.79)**
	Citalopram vs. Venlafaxine	**0.49 (0.34, 0.72)**
	Citalopram vs. Fluoxetine	0.86 (0.57, 1.29)
	Citalopram vs. Mirtazapine	0.64 (0.40, 1.03)
	Citalopram vs. Duloxetine	1.09 (0.51, 2.31)
	Citalopram vs. Quetiapine	**0.47 (0.30, 0.76)**
	Escitalopram vs. Sertraline	**0.47 (0.31, 0.72)**
	Escitalopram vs. Venlafaxine	**0.43 (0.28, 0.65)**
	Escitalopram vs. Fluoxetine	0.75 (0.48, 1.17)
	Escitalopram vs. Mirtazapine	**0.56 (0.34, 0.93)**
	Escitalopram vs. Duloxetine	0.95 (0.44, 2.06)
	Escitalopram vs. Quetiapine	**0.42 (0.25, 0.69)**
	Sertraline vs. Venlafaxine	0.91 (0.61, 1.36)
	Sertraline vs. Fluoxetine	**1.59 (1.03, 2.44)**
	Sertraline vs. Mirtazapine	1.19 (0.73, 1.94)
	Sertraline vs. Duloxetine	2.00 (0.93, 4.32)
	Sertraline vs. Quetiapine	0.87 (0.53, 1.44)
	Venlafaxine vs. Fluoxetine	**1.74 (1.13, 2.69)**
	Venlafaxine vs. Mirtazapine	1.31 (0.80, 2.14)
	Venlafaxine vs. Duloxetine	**2.20 (1.02, 4.75)**
	Venlafaxine vs. Quetiapine	0.96 (0.59, 1.58)
	Fluoxetine vs. Mirtazapine	0.75 (0.45, 1.26)

	Fluoxetine vs. Duloxetine	1.26 (0.58, 2.77)
	Fluoxetine vs. Quetiapine	**0.55 (0.33, 0.93)**
	Mirtazapine vs. Duloxetine	1.68 (0.74, 3.82)
	Mirtazapine vs. Quetiapine	0.74 (0.41, 1.31)
	Duloxetine vs. Quetiapine	**0.44 (0.19,0.99)**
Atrial Fibrillation	Citalopram vs.Escitalopram	0.76(0.36,1.58)
	Citalopram vs. Sertraline	0.95(0.47,1.90)
	Citalopram vs. Venlafaxine	0.56(0.25,1.24)
	Citalopram vs. Fluoxetine	2.15(0.94,4.91)
	Citalopram vs. Mirtazapine	1.02(0.36,2.90)
	Citalopram vs. Duloxetine	0.75(0.37,1.54)
	Citalopram vs. Quetiapine	0.56(0.12,2.59)
	Escitalopram vs. Sertraline	1.26(0.63,2.49)
	Escitalopram vs. Venlafaxine	0.74(0.34,1.62)
	Escitalopram vs. Fluoxetine	**2.84(1.25,6.44)**
	Escitalopram vs. Mirtazapine	1.35(0.48,3.81)
	Escitalopram vs. Duloxetine	0.99(0.49,2.02)
	Escitalopram vs. Quetiapine	0.75(0.16,3.41)
	Sertraline vs. Venlafaxine	0.59(0.28,1.24)
	Sertraline vs. Fluoxetine	**2.26(1.04,4.92)**
	Sertraline vs. Mirtazapine	1.07(0.39,2.94)
	Sertraline vs. Duloxetine	0.79(0.41,1.53)
	Sertraline vs. Quetiapine	0.59(0.13,2.65)
	Venlafaxine vs. Fluoxetine	**3.83(1.61,9.12)**
	Venlafaxine vs. Mirtazapine	1.82(0.62,5.33)
	Venlafaxine vs. Duloxetine	1.34(0.62,2.87)

	Venlafaxine vs. Quetiapine	1.00(0.21,4.71)
	Fluoxetine vs. Mirtazapine	0.48(0.16,1.43)
	Fluoxetine vs. Duloxetine	**0.35(0.16,0.78)**
	Fluoxetine vs. Quetiapine	0.26(0.06,1.25)
	Mirtazapine vs. Duloxetine	0.74(0.26,2.05)
	Mirtazapine vs. Quetiapine	0.55(0.10,2.99)
	Duloxetine vs. Quetiapine	0.75(0.17,3.40)
Heart Block	Citalopram vs.Escitalopram	**4.35(1.83,10.33)**
	Citalopram vs. Sertraline	**2.39(1.13,5.05)**
	Citalopram vs. Venlafaxine	**22.97(6.03,87.60)**
	Citalopram vs. Fluoxetine	0.80(0.32,2.02)
	Citalopram vs. Mirtazapine	**7.19(2.38,21.68)**
	Citalopram vs. Duloxetine	**10.69(1.13,101.32)**
	Citalopram vs. Quetiapine	1.11(0.45,2.75)
	Escitalopram vs. Sertraline	0.55(0.25,1.19)
	Escitalopram vs. Venlafaxine	**5.28(1.38,20.24)**
	Escitalopram vs. Fluoxetine	**0.18(0.07,0.48)**
	Escitalopram vs. Mirtazapine	1.65(0.54,5.03)
	Escitalopram vs. Duloxetine	2.46(0.26,23.38)
	Escitalopram vs. Quetiapine	**0.26(0.10,0.65)**
	Sertraline vs. Venlafaxine	**9.62(2.68,34.52)**
	Sertraline vs. Fluoxetine	**0.33(0.14,0.79)**
	Sertraline vs. Mirtazapine	**3.01(1.07,8.44)**
	Sertraline vs. Duloxetine	4.47(0.49,40.96)
	Sertraline vs. Quetiapine	0.47(0.20,1.07)
	Venlafaxine vs. Fluoxetine	**0.04(0.01,0.14)**

	Venlafaxine vs. Mirtazapine	0.31(0.07,1.41)
	Venlafaxine vs. Duloxetine	0.47(0.04,5.51)
	Venlafaxine vs. Quetiapine	**0.05(0.01,0.19)**
	Fluoxetine vs. Mirtazapine	**9.02(2.76,29.42)**
	Fluoxetine vs. Duloxetine	**13.40(1.36,132.18)**
	Fluoxetine vs. Quetiapine	1.39(0.51,3.79)
	Mirtazapine vs. Duloxetine	1.49(0.14,15.64)
	Mirtazapine vs. Quetiapine	**0.16(0.05,0.50)**
	Duloxetine vs. Quetiapine	0.10(0.01,1.02)
Ventricular Arrhythmia	Citalopram vs.Escitalopram	**3.88(1.68,8.95)**
	Citalopram vs. Sertraline	1.43(0.70,2.92)
	Citalopram vs. Venlafaxine	1.02(0.52,1.98)
	Citalopram vs. Fluoxetine	1.06(0.42,2.64)
	Citalopram vs. Mirtazapine	0.67(0.24,1.85)
	Citalopram vs. Duloxetine	1.62(0.49,5.36)
	Citalopram vs. Quetiapine	**0.44(0.21,0.92)**
	Escitalopram vs. Sertraline	**0.37(0.16,0.86)**
	Escitalopram vs. Venlafaxine	**0.26(0.12,0.59)**
	Escitalopram vs. Fluoxetine	**0.27(0.10,0.75)**
	Escitalopram vs. Mirtazapine	**0.17(0.06,0.53)**
	Escitalopram vs. Duloxetine	0.42(0.12,1.50)
	Escitalopram vs. Quetiapine	**0.11(0.05,0.27)**
	Sertraline vs. Venlafaxine	0.71(0.36,1.41)
	Sertraline vs. Fluoxetine	0.74(0.29,1.87)
	Sertraline vs. Mirtazapine	0.47(0.17,1.31)
	Sertraline vs. Duloxetine	1.14(0.34,3.79)

Sertraline vs. Quetiapine	**0.31(0.15,0.65)**
Venlafaxine vs. Fluoxetine	1.04(0.43,2.52)
Venlafaxine vs. Mirtazapine	0.66(0.25,1.78)
Venlafaxine vs. Duloxetine	1.60(0.49,5.16)
Venlafaxine vs. Quetiapine	**0.43(0.21,0.88)**
Fluoxetine vs. Mirtazapine	0.64(0.20,2.05)
Fluoxetine vs. Duloxetine	1.54(0.41,5.81)
Fluoxetine vs. Quetiapine	0.42(0.16,1.07)
Mirtazapine vs. Duloxetine	2.41(0.60,9.78)
Mirtazapine vs. Quetiapine	0.65(0.23,1.83)
Duloxetine vs. Quetiapine	**0.27(0.08,0.91)**
